# A 39‐Year Nationwide Study of Uveal Melanoma in Taiwan

**DOI:** 10.1002/cam4.70754

**Published:** 2025-04-15

**Authors:** An‐Ning Chao, Angel Chao, Wei‐Yang Chang, Chun‐Chieh Wang, Yueh‐Ju Tsai, Kuan‐Jen Chen, Yih‐Shiou Hwang, Lan‐Yan Yang

**Affiliations:** ^1^ Department of Ophthalmology Linkou Chang Gung Memorial Hospital and Chang Gung University College of Medicine Taoyuan Taiwan; ^2^ Department of Obstetrics and Gynecology Linkou Chang Gung Memorial Hospital and Chang Gung University College of Medicine Taoyuan Taiwan; ^3^ Gynecologic Cancer Research Center Linkou Chang Gung Memorial Hospital and Chang Gung University College of Medicine Taoyuan Taiwan; ^4^ Biostatistics Unit of Clinical Trial Center Chang Gung Memorial Hospital Taiwan; ^5^ Department of Radiation Oncology Linkou Chang Gung Memorial Hospital and Chang Gung University College of Medicine Taoyuan Taiwan; ^6^ Division of Clinical Trial, Department of Medical Research Taichung Veterans General Hospital Taichung Taiwan

**Keywords:** incidence, nationwide study, survival, Taiwan, treatment, uveal melanoma

## Abstract

**Purpose:**

To investigate the incidence, treatment strategies, and survival outcomes of adult patients with uveal melanoma (UM) in Taiwan over a 39‐year period.

**Design:**

We conducted a retrospective, nationwide population‐based cohort study retrieving data from the Taiwan Cancer Registry and the National Death Registry of Health and Welfare Data Science Center database. By employing the International Classification of Diseases diagnostic codes, we identified patients who were diagnosed with UM between January 1980 and December 2018. We analyzed treatment information, as well as mortality data, to gain a comprehensive understanding of the incidence, patient characteristics, treatment patterns, and survival outcomes.

**Results:**

A total of 314 patients (156 males and 158 females) were diagnosed with UM. The overall incidence rate was 0.36 (range: 0.30–0.42) per million persons, with a mean age at diagnosis of 52 years. In terms of treatment options, enucleation was performed on 122 patients (38.9%), whereas 108 (34.4%) received radiotherapy (RT). The remaining 84 (26.8%) patients underwent alternative treatments. The 5‐year overall survival (OS) rate was 58.3%. Pairwise comparisons showed that the OS rates of patients who underwent RT *versus* surgery were similar. Two factors were associated with favorable OS outcomes: being under 50 years of age (*p* < 0.001) and being a female (*p* = 0.04).

**Conclusions:**

Our real‐world study encompassing a 39‐year timeframe revealed a relatively low incidence of UM within the Taiwanese population. Patients under the age of 50, as well as females, demonstrated more favorable OS outcomes.

## 1 Introduction

Uveal melanoma (UM) stands as the most common primary intraocular cancer among adults, with varying incidence rates across different geographic regions and ethnic groups [1, 2, 3, 4]. Although this malignancy predominantly impacts Caucasian individuals, the extent to which UM affects various populations worldwide has not been entirely elucidated. In the United States, the Surveillance, Epidemiology, and End Results (SEER) study found an incidence rate of 5.1 cases per million people [5, 6]. European countries display varying incidence rates, ranging from 2 to 8 cases per million, with a noticeable increase from Southern to Northern Europe [7, 8]. Information on UM in the Asia‐Pacific region is limited; however, current research indicates that the annual age‐adjusted incidence rate ranges between 0.25 and 0.6 cases per million population [9, 10, 11, 12, 13]. This evidence demonstrates a lower epidemiological impact of this malignancy among Asian communities compared to their Western counterparts. Comprehensive assessments conducted in Asia have the potential to not only establish a robust foundation for advancing clinical care but also to enhance prognostic outcomes for individuals affected by this neoplasm, tailored to the specific needs and characteristics of the region.

During the late 19th century, enucleation was the standard treatment for UM. However, the late 1970s marked the emergence of eye‐preserving therapies, significantly broadening treatment options. Currently, UM management includes various modalities such as enucleation, local plaque brachytherapy, external beam fractionated stereotactic radiotherapy (RT), proton beam RT, and transpupillary thermotherapy (TTT), which is specifically designed for small tumors [14, 15, 16]. However, TTT—a laser‐based technique—is now primarily used as an adjunctive therapy due to its high rates of local and orbital recurrence when applied as a standalone treatment. Unfortunately, despite advancements in diagnostic methods and the adoption of globe‐sparing therapies [5, 17, 18], the 5‐year overall survival (OS) rates for patients with UM in the Asian population remain disappointingly stagnant.

In this retrospective, nationwide real‐world study, we aimed to enhance our understanding of the long‐term trends in incidence, treatment approaches, and survival outcomes for adult Taiwanese patients with UM. By analyzing these factors over a 39‐year period, we utilized the comprehensive population‐based data from the Taiwan Cancer Registry (TCR) housed within the Health and Welfare Data Science Center. This extensive dataset provides detailed insights into healthcare utilization patterns, enabling us to identify areas for improvement in the real‐world management of UM. Our analysis aimed to lay the groundwork for developing more effective strategies to address this intraocular malignancy within the Asian context, ultimately contributing to enhanced patient outcomes and care.

## 2 Methods

### 2.1 Ethics

The research protocol was approved by both the Health and Welfare Data Science Center of the TCR and the Institutional Review Board of Chang Gung Memorial Hospital (Reference Number: 202200384B0). Given the retrospective nature of the investigation and its reliance on secondary data analysis, the need for informed consent was waived. To protect participant privacy, all individual data were thoroughly anonymized prior to analysis. To further safeguard confidentiality, estimates were not reported when the number of cases was less than three.

### 2.2 Data Sources

**FIGURE 1:**
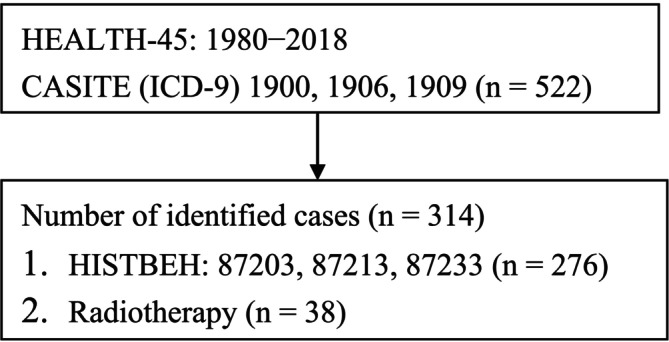
Study flowchart. The category of Taiwan's National Health Insurance Research Database is represented by HEALTH‐45. Abbreviations: CASITE, cancer site; HISTBEH, histological behavior; ICD‐9, ninth version of the International Classification of Diseases.

The study complied with the principles set forth in the Declaration of Helsinki and used data from all eligible patients obtained from the nationwide TCR database (Health and Welfare Data Science Center, Ministry of Health and Welfare, Taiwan; Figure 1). The TCR was first established in 1979 and prospectively recorded information on all patients with malignancies in Taiwan—including site‐specific variables and other clinical parameters related to patient care. As of 2005, the reported completeness of data registration in the TCR ranged between 97% and 98.4% [19]. The assessment and review of catastrophic illness status applications by primary care physicians began in 2001 under the supervision of the Bureau of National Health Insurance. This study included comprehensive data sets spanning 1980–2018, which included demographic data, diagnostic information, prescription records, outpatient and inpatient treatment modalities, and updated registries for individuals with catastrophic illness status. In Taiwan, UM is classified as a catastrophic illness. Primary care physicians and ophthalmologists evaluated the eligibility for this status, ensuring the accuracy of patient information. Furthermore, mortality data were corroborated using cancer registry records and catastrophic illness certificates.

### 2.3 Study Patients

Eligibility criteria for this study comprised patients diagnosed with UM who were included in the TCR, identified through specific ICD‐9 diagnostic codes, including 190.0 (malignant neoplasm of the eyeball), 190.6 (malignant neoplasm of the choroid), and 190.9 (malignant neoplasm of the eye). UM included the following histological types: 87203 (malignant melanoma), 87,213 (nodular melanoma), and 87,233 (malignant melanoma in regression). The study cohort consisted of patients diagnosed between January 1980 and December 2018. All‐cause mortality data were retrieved from the Taiwanese National Death Registry (NDR) of the Department of Health [20]. OS was defined as the time interval from the UM diagnosis to death from any cause or censored at the last follow‐up. Follow‐up was terminated on December 31, 2018.

### 2.4 Statistical Analysis

Continuous variables were described using their mean, standard deviation (SD), median, and range (minimum to maximum values). To compare continuous variables across groups, we applied either the independent Student's *t*‐test or the Wilcoxon rank‐sum test, depending on the distribution of the data. Categorical variables were analyzed using the chi‐square test. Furthermore, pairwise comparisons of RT and surgical procedures were performed, with Bonferroni correction applied to mitigate potential bias arising from multiple comparisons. To estimate survival curves, we generated Kaplan–Meier plots and assessed differences between the groups utilizing the log‐rank test. Both univariable and multivariable Cox regression analyses were employed to evaluate the relationships between the study variables and OS. For every variable considered, the estimated hazard ratios and their corresponding 95% confidence intervals (CIs) were calculated. Analyses were performed using the SAS software package, version 9.4 (SAS Institute Inc., Cary, NC, USA). Two‐tailed *p*‐values < 0.05 were considered statistically significant.

## 3 Results

### 3.1 Incidence of Uveal Melanoma

**FIGURE 2:**
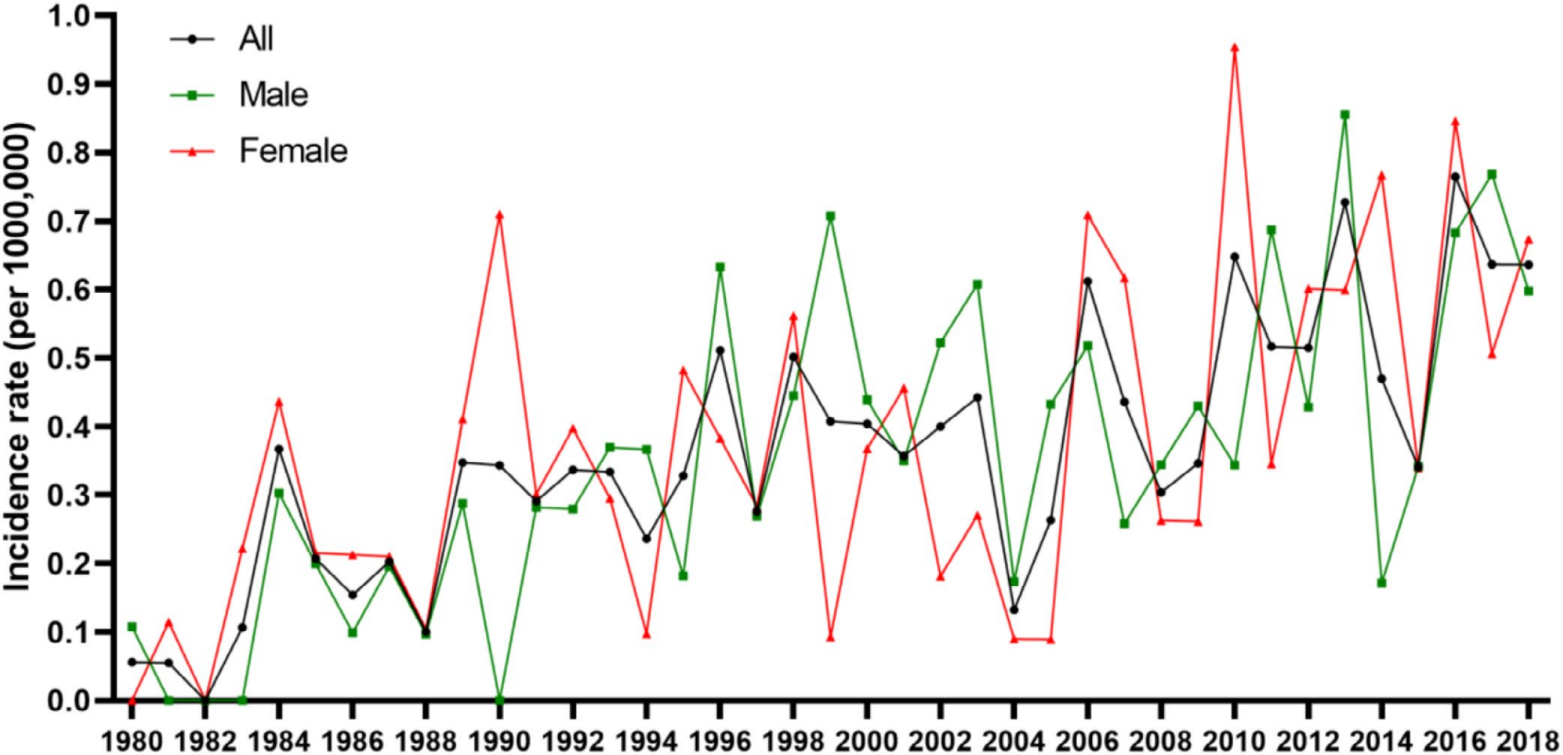
Annual incidence of uveal melanoma in Taiwan between 1980 and 2018.

Between 1980 and 2018, 314 adult patients (156 males and 158 females) with UM were identified within the Taiwanese population (Table 1). The overall incidence rate was 0.36 (range: 0.30–0.42) per million persons (Figure 2). A sex‐based evaluation showed an incidence rate of 0.35 (range: 0.28–0.43) per million males, whereas women exhibited a slightly higher rate of 0.37 (range: 0.29–0.45) per million females. The mean age at diagnosis was 51.9 years (Table 1). In addition, the highest occurrence of UM was observed in individuals belonging to the ≥ 60 years age group. Overall, no statistically significant sex‐related difference was detected in the age at diagnosis. However, a consistent upward trend in the incidence of UM was noted (Figure 2).

**TABLE 1:** Sex‐specific differences in Taiwanese patients with uveal melanoma.

Characteristic	Entire cohort (*n* = 314)		Males (*n* = 156)		Females (*n* = 158)		*p* *
	*n*	%	*n*	%	*n*	%	
Age, years; mean (SD)	51.9 (17.1)		52.8 (16.5)		50.9 (17.6)		0.322
Age category, years							0.067
< 50	137	(43.6)	60	(38.5)	77	(48.7)	
≥ 50	177	(56.4)	96	(61.5)	81	(51.3)	
Age category, years^a^							0.461
0–18	8	(2.5)	5	(3.2)	3	(1.9)	
19–29	20	(6.4)	9	(5.8)	11	(7.0)	
30–39	46	(14.6)	19	(12.2)	27	(17.1)	
40–49	63	(20.1)	27	(17.3)	36	(22.8)	
50–59	67	(21.3)	36	(23.1)	31	(19.6)	
≥ 60	110	(35)	60	(38.5)	50	(31.6)	
Treatment modality							0.229
Radiotherapy	108	(34.4)	49	(31.4)	59	(37.3)	
Surgery	122	(38.9)	68	(43.6)	54	(34.2)	
Other treatments^a^	84	(26.8)	39	(25)	45	(28.5)	

Abbreviation: SD, standard deviation.

^a^ Include treatment with laser as well as missing data for self‐paid therapeutic modalities not documented in the National Health Insurance Research Database. Variables are reported as counts and percentages unless specified otherwise.

* *p*‐Values were calculated using the Student's *t*‐test, Wilcoxon rank‐sum test, or chi‐squared test, as appropriate.

### 3.2 Treatment Strategies

Of the 314 patients included in the study, 122 (38.9%) were treated with enucleation, while 108 (34.4%) underwent RT, specifically fractionated stereotactic RT. The remaining 84 patients (26.8%) received alternative treatment modalities, including laser therapy, or opted for treatments outside the purview of the National Health Insurance Program, which were self‐financed and therefore not captured within the health scheme's documentation (Table 1). No distinct treatment patterns were found across different age groups (*p* = 0.324) (Table 2). Throughout the study period, a notable trend emerged, characterized by an increase in the utilization of RT, while other treatment modalities exhibited a corresponding decline (*p* < 0.001, Table 3).

**TABLE 2:** Treatment‐specific differences in Taiwanese patients with uveal melanoma by age groups.

Characteristics	Radiotherapy (*n* = 108)		Surgery (*n* = 122)		Other treatments (*n* = 84)		*p*
	*N*	%	*N*	%	*N*	%	
Age, years							0.324
< 50	41	(38)	58	(47.5)	38	(45.2)	
≥ 50	67	(62)	64	(52.5)	46	(54.8)	
Age category, years							0.188
0–29^a^	6	(5.6)	11	(9.0)	11	(13.1)	
30–39	11	(10.2)	22	(18.0)	13	(15.5)	
40–49	24	(22.2)	25	(20.5)	14	(16.7)	
50–59	28	(25.9)	18	(14.8)	21	(25.0)	
≥ 60	39	(36.1)	46	(37.7)	25	(29.7)	

*Note:* Data are reported as counts and percentages. The chi‐squared test was employed to calculate *p*‐values.

^a^ To protect the privacy of the study participants, estimates were not reported when the number of cases were fewer than three. Age groups 0–18 and 19–29 were combined.

**TABLE 3:** Incidence of uveal melanoma in Taiwan (1980–2018) categorized by distinct study periods.

Characteristic	1980–1989 (*n* = 31)		1990–1999 (*n* = 76)		2000–2009 (*n* = 84)		2010–2018 (*n* = 123)		*p*
	*n*	%	*n*	%	*n*	%	*n*	%	
Age category, years									0.027
< 50	16	(51.6)	40	(52.6)	40	(47.6)	41	(33.3)	
≥ 50	15	(48.4)	36	(47.4)	44	(52.4)	82	(66.7)	
Sex									0.441
Male	13	(41.9)	39	(51.3)	47	(56)	57	(46.3)	
Female	18	(58.1)	37	(48.7)	37	(44)	66	(53.7)	
Treatment modality									< 0.001
Radiotherapy	3	(9.7)	11	(14.5)	25	(29.8)	69	(56.1)	
Surgery or other treatments	28	(90.3)	65	(85.5)	59	(70.2)	54	(43.9)	

*Note:* Data are reported as counts and percentages. The chi‐squared test was employed to calculate *p*‐values.

### 3.3 Survival outcomes

**FIGURE 3:**
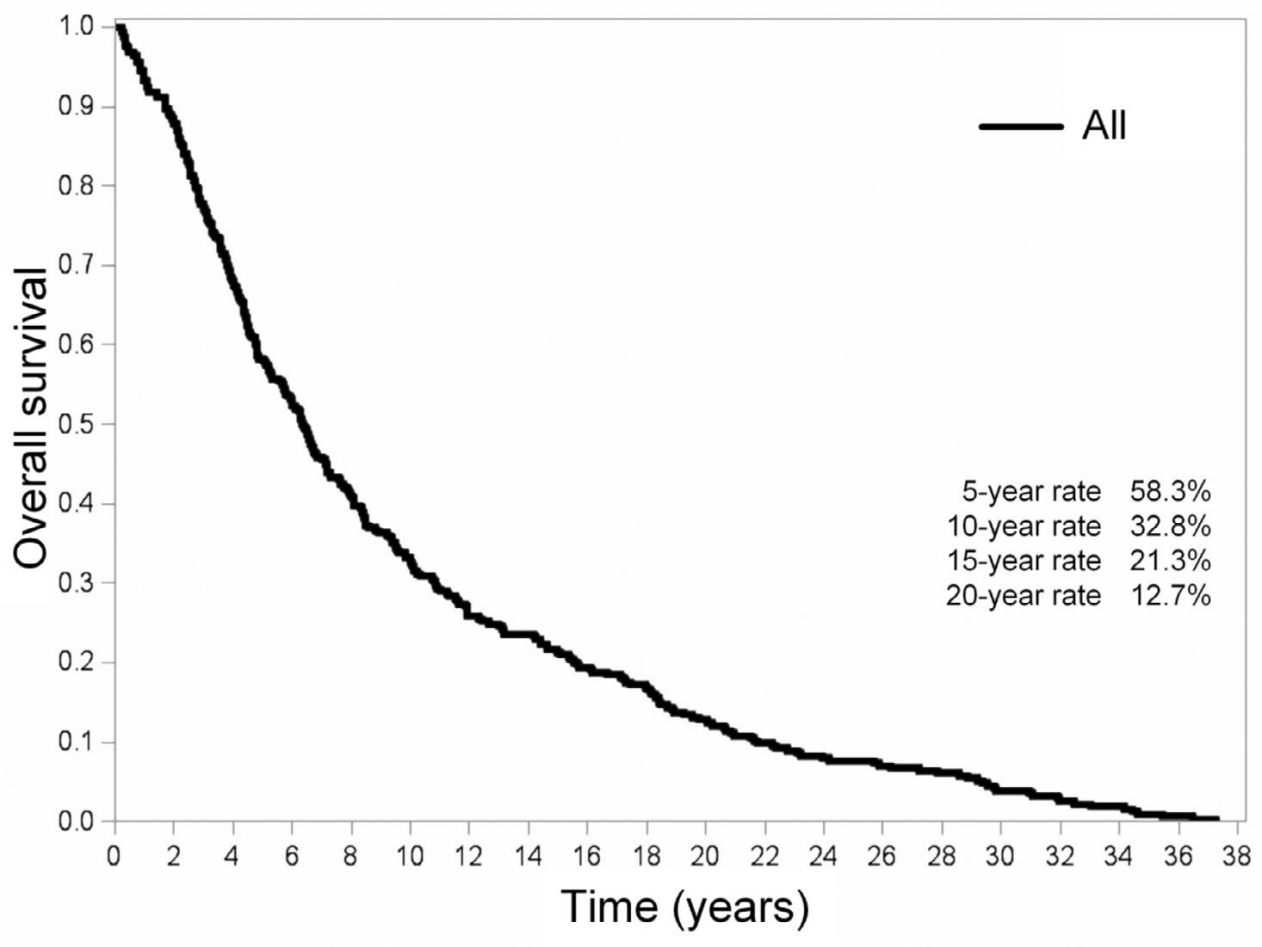
Kaplan–Meier plot illustrating the overall survival for Taiwanese patients with uveal melanoma diagnosed between 1980 and 2018.

**FIGURE 4:**
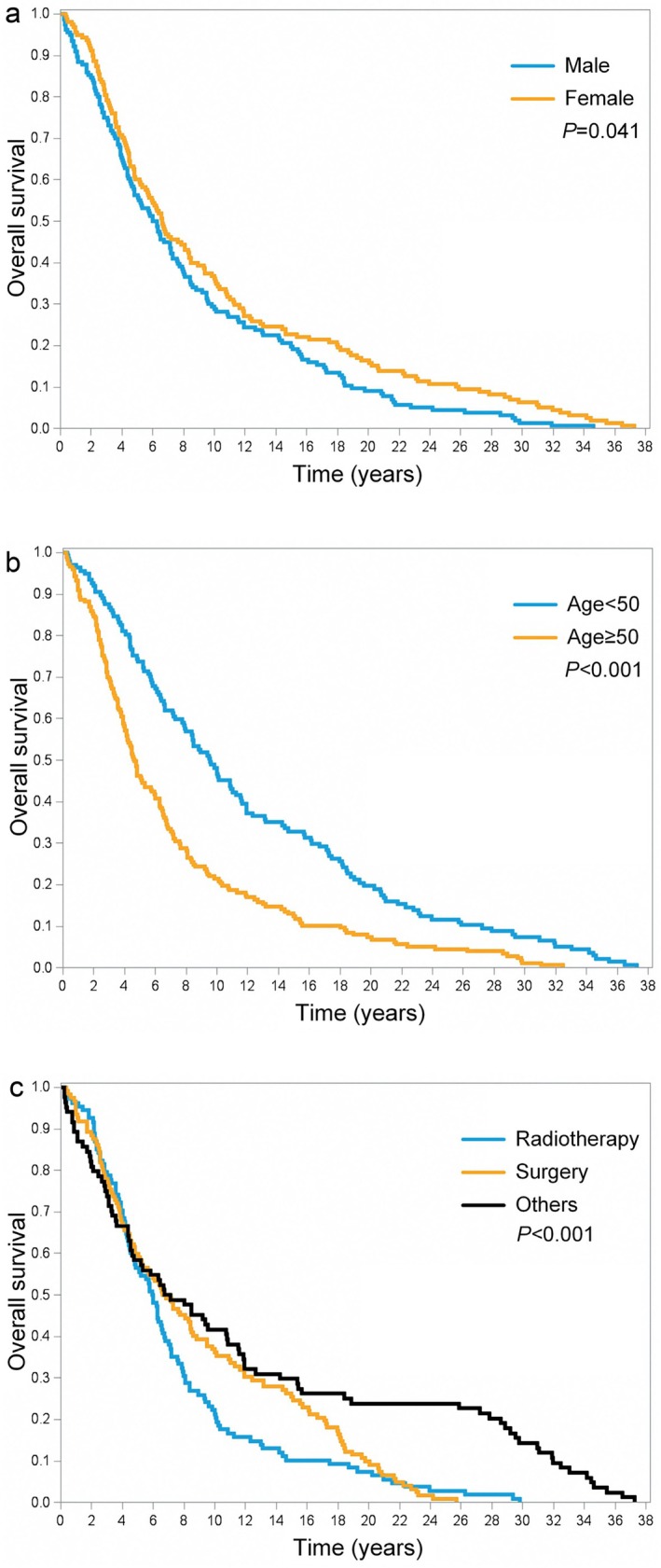
(a) Kaplan–Meier plots illustrating the sex‐based differences in overall survival for Taiwanese patients with uveal melanoma diagnosed between 1980 and 2018. (b) Kaplan–Meier plots illustrating the age‐based differences in overall survival for Taiwanese patients with uveal melanoma diagnosed between 1980 and 2018. (c) Kaplan–Meier plots illustrating the treatment‐based differences in overall survival for Taiwanese patients with uveal melanoma diagnosed between 1980 and 2018.

The 5‐, 10‐, 15‐, and 20‐year OS rates for Taiwanese patients diagnosed with UM were 58.3%, 32.8%, 21.3%, and 12.7%, respectively (Figure 3). Interestingly, males displayed a significantly lower OS compared to females (*p* = 0.041; Figure 4a). However, patients who were below 50 years of age demonstrated more favorable OS outcomes compared to their counterparts aged 50 and above (*p* < 0.001; Figure 4b). Our analysis further highlighted a significant impact of treatment type on OS outcomes (*p* < 0.001; Figure 4c). However, upon pairwise comparison, the OS rates between patients who underwent RT versus surgery did not exhibit any significant difference (*p* = 0.742). We identified two factors independently associated with more favorable OS outcomes: being under 50 years of age (*p* < 0.001) and being female (*p* = 0.041) (Table 4).

**TABLE 4:** Predictors of overall survival in Taiwanese patients with uveal melanoma: univariable and multivariable analyses.

	Univariable analysis				Multivariable analysis		
	*n*	Hazard ratio	95% confidence interval	*p*	Hazard ratio	95% confidence interval	*p*
Age category, years							
< 50	137	Reference			Reference		
≥ 50	177	1.86	1.48–2.34	< 0.001	1.83	1.45–2.3	< 0.001
Sex							
Male	156	Reference			Reference		
Female	158	0.79	0.63–0.99	0.041	0.88	0.7–1.1	0.255
Treatment modality							
Radiotherapy	108	Reference			Reference		
Surgery	122	0.83	0.64–1.08	0.169	0.87	0.67–1.14	0.312
Other treatments	84	0.52	0.38–0.72	< 0.001	0.55	0.40–0.75	< 0.001

## 4 Discussion

Our study revealed three primary findings. Firstly, the overall incidence rate of UM in Taiwan from 1980 to 2018 was found to be relatively low, at 0.36 (range: 0.29–0.42) per million persons; however, there was a steady increase in the incidence of this malignancy during the study period. Secondly, enucleation and RT emerged as the most prevalent treatment methods, accounting for 38.5% and 35.3% of cases, respectively. No discernible age‐related differences in treatment preferences were observed. Lastly, the 5‐, 10‐, 15‐, and 20‐year OS rates for Taiwanese patients diagnosed with UM stood at 58%, 32.5%, 20.5%, and 12%, respectively. Notably, we identified two factors independently correlated with more favorable OS outcomes, that is, being under 50 years of age and being female.

The observed incidence of UM in Taiwan, a country predominantly inhabited by mono‐ethnic Chinese individuals accounting for 90% of the population, was notably lower in comparison to the United States and Europe. However, our epidemiological observations are consistent with patterns seen in other Mongoloid Asian populations, such as Chinese, Japanese, and Korean groups, which display rates between 0.25 and 0.6 cases per million people [10, 11, 12, 13]. Regarding temporal patterns, we observed a nonsignificant trend of raising incidence of UM from 1980 to 2018. A comparable observation was documented by Park et al. [12], who identified an escalating incidence of this malignancy between 2006 and 2011. We hypothesize that the observed upward trend in our study may partly reflect the increasing proportion of elderly individuals within Taiwan's population.

The observed epidemiological changes may be attributed to dynamic shifts in the country's demographic structure (Figure 2). According to data from Taiwan's National Health Research Institutes, there has been a marked demographic transformation over the recent decades. In 1989, the population of individuals aged 50 or older was 3,510,073, whereas by 2018, this number had increased substantially to 8,695,802. In contrast, the population of individuals under 50 years of age demonstrated a decreasing trend over the same period. Specifically, in 1989, this demographic group consisted of 16,646,514 individuals, which decreased to 14,893,130 by 2018.

No statistically significant sex‐related differences were observed in the occurrence of UM. This finding aligns with data from patient groups in Japan, Korea, and China, which also reported an equivalent prevalence of this malignancy in both sexes [10, 12, 21]. Riazi‐Esfahani and colleagues [22] found no sex differences in UM regarding tumor thickness, diameter, or patient age in the Middle East. However, a study by Singh et al. [6] presented contrasting findings, highlighting a sex‐related disparity in UM incidence within the United States. Specifically, between 1973 and 2008, male patients exhibited a significantly higher age‐adjusted incidence rate of 5.8 per million, compared to females with an average age‐adjusted rate of 4.4 per million people [6]. Similarly, in Europe, the incidence rate has been reported to be higher in males than in females [8, 23, 24].

Our study found that the mean age of patients with UM was 52 years. The median age at diagnosis in our study was younger than that observed in the United States (62 years) [6] and Japan (63 years) [13], but aligned more closely with the median age observed in Korea (54 years) [12]. However, it was notably higher than the median age reported in China (44 years) [25]. These findings collectively indicate that individuals of Han ethnicity tend to develop UM at a younger age compared to other ethnic groups.

Despite its widespread global adoption, plaque brachytherapy is not available in Taiwan, primarily due to the relatively low incidence of UM in the region, which poses significant challenges in establishing and sustaining this specialized treatment modality. In the current study, enucleation was the primary treatment modality, followed by external beam RT—including stereotactic and gamma knife techniques—and other treatment strategies such as laser therapy, brachytherapy, and proton beam therapy performed abroad. Notably, patients who underwent alternative treatments demonstrated the highest survival rate. However, definitive conclusions regarding treatment outcomes are precluded by the absence of tumor thickness data in our dataset, underscoring the need for comprehensive clinical information to accurately compare therapeutic efficacy. While the National Health Insurance Program guarantees mandatory health insurance coverage for over 98% of the population, offering a broad spectrum of healthcare services, it does not currently include reimbursements for proton beam therapy. The 5‐year OS rate observed in our study aligns with the 53% survival outcome reported for a cohort of 33 Taiwanese patients, of which 76% (26 patients) underwent enucleation in 2013 [25]. Additionally, this rate parallels the 61% survival rate found in a small cohort of 72 patients who received enucleation treatment in Taiwan [26]. However, the survival rate in our study was lower relative to other investigations conducted in Western countries. These include the EUROCARE study (68.9%) [18], a study from Denmark (66% in males and 69% in females) [27], and a research from the United States (80%) [5].

In our cohort, we observed that females exhibited significantly more favorable OS outcomes than males. While the Collaborative Ocular Melanoma Study did not report sex differences in terms of survival [14], the EUROCARE study found a 10% increased mortality rate among male patients [18]. Slightly improved survival outcomes for female patients have also been documented in studies conducted in Sweden, Denmark, and the United Kingdom [18]. The poorer survival outcomes observed in male patients with UM are strongly associated with factors such as larger tumor size, monosomy 3, and chromosome 8q gains. These variables can be effectively assessed using competing risk analyses [28]. Additionally, accurate estimation of tumor volume may refine prognostication in cases involving metastatic lesions.

The strengths of this study are rooted in its nationwide population‐based design and the extensive observation period. With more than 98% of Taiwan's population covered by the National Health Insurance program, both the incidence and OS outcomes were accurately estimated, despite minor data entry errors. Nonetheless, a small number of false‐negative cases in treatment may exist, primarily due to some patients refusing to apply for medical co‐payment waivers. This issue is partially offset by linking data from the National Cancer Registry, resulting in a positive predictive value of 94% for cancer diagnosis within the National Health Insurance dataset [29]. Despite the potential for bias due to misclassification or under detection, the TCR has effectively allowed us to assess the incidence, treatment modalities, and survival outcomes of UM in our country. However, it is important to note that the TCR does not include specific parameters such as tumor size, histopathological characteristics, or visual acuity, as these variables are not captured within the registry. We also recognize the significance of assessing local recurrence rates—especially in relation to TTT. Regrettably, our data sources did not include records of local recurrence rates. A prior study demonstrated a significant correlation between tumor size and older age with survival outcomes, providing robust estimates for all‐cause mortality following enucleation [30]. To further elucidate long‐term outcomes and the prognostic implications of unquantified variables, it is essential to conduct large‐scale, comprehensive investigations. These studies should prioritize extended follow‐up periods, detailed tumor data, visual prognosis, and a thorough evaluation of the efficacy and complications associated with various treatment modalities.

## 5 Conclusions

Our investigation into UM among Taiwanese adults revealed a notably low incidence. Interestingly, patients under 50 years of age and females demonstrated significantly better OS. In addition, we observed comparable OS rates between patients who underwent RT and those who received surgical intervention. These findings may provide valuable insights into UM epidemiology and treatment outcomes in Taiwan, potentially guiding future clinical practices and research endeavors.

## Author Contributions

**An‐Ning Chao:** conceptualization (lead), data curation, investigation, methodology, resources, writing – original draft, writing – review and editing (lead). **Angel Chao:** conceptualization, funding acquisition, investigation, project administration, supervision,writing – review and editing. **Wei‐Yang Chang:** data curation, formal analysis, resources, software, Validation. **Chun‐Chieh Wang:** data curation, resources, writing – review and editing. **Yueh‐Ju Tsai:** data curation, investigation; resources. **Kuan‐Jen Chen:** data curation, resources, writing – review and editing. **Yih‐Shiou Hwang:** data curation, resources, writing – review and editing. **Lan‐Yan Yang:** data curation (lead), formal analysis, investigation, methodology, resources, software, writing – original draft.

## Conflicts of Interest

The authors declare no conflicts of interest.

## Data Availability

Data that support the findings of this study will be provided from the corresponding author upon reasonable request.
